# A “Healthy” Color: Information About Healthy Eating Attenuates the “Red Effect”

**DOI:** 10.5539/gjhs.v8n1p56

**Published:** 2015-05-15

**Authors:** Alyssa Gilston, Gregory J. Privitera

**Affiliations:** 1 School of Advanced Studies, University of Phoenix, Tempe, AZ, USA

**Keywords:** red effect, color, attractiveness

## Abstract

The influence of the color red on our evaluative processes and psychological, emotional, and cognitive decision making is known as the “red effect.” The present study tested the hypothesis that this “red effect” could be attenuated by information about the healthfulness of an individual’s diet. To test this hypothesis 122 participants rated the attractiveness and healthfulness of a picture of the same female wearing a red or white (neutral color) shirt who was described as eating a healthy or unhealthy food. Results showed that participants did rate the female wearing the red shirt as more attractive (evidence of the “red effect”). However, when the female was described as eating an unhealthy food, the red effect was not significant. These findings suggest that the red effect is robust, but can be attenuated by manipulating the perceptions of health.

## 1. Introduction

The concept of color as it relates to motivation, decision making and perception has been widely explored. Research supports the idea that color can and does impact perception (Puccinelli, Chandrasekaran, Grewal, & Suri, 2013). Color research dates back decades and several studies have demonstrated that colors can impact mood (Valdez & Mehrabian, 1994). Color conveys information that extends beyond appearance and aesthetics, and has been shown to contain emotional meaning and associations (Gil & LeBigot, 2014). There is evidence that color may impact our judgment and choices, and a majority of studies related to color research focus on the color red. Past research indicates that the color red can also affect psychological functioning (Zhang & Han, 2014). Different colors have different associations, and people tend to develop these associations when they consistently encounter scenarios where a specific color is accompanied by particular experiences (Mehta & Zhu, 2009). This influence of the color red on our psychological functioning and, emotional and cognitive decision making is known as the *red effect*.

Human beings perceive a spectrum of color mostly because objects can selectively absorb some wavelengths of light while reflecting others (Schacter, Gilbert, & Wegner, 2009). Sir Isaac Newton is credited with discovering the fact that seeing color is done by our perception of light’s wavelength. We perceive the shortest visible wavelength as deep purple, and as wavelengths increase, the color perceived will gradually and continuously change from blue, to green, then yellow, orange and finally red, which has the longest visible wavelength (Schacter et al., 2009). We tend to identify three primary colors as red, blue and yellow, and each color is commonly associated with different physical and psychological attributes.

According to Elliot and Maier (2014), the color-in-context theory suggests that color automatically activates a variety of evaluative processes which influence psychological functioning. Recent research on the color red has indicated that red is associated with failure, threat, and also reward (Buechner, Maier, Lichtenfeld, & Schwarz, 2014). Buechner et al. (2014), suggested that the color red functions as a signal of increased relevance and that it also strengthens processing of some goal-related stimuli. They further examined the combined influence of expression of emotion and color on increased attention, and found that red increases attentional engagement reward (Buechner et al., 2014).

Zhang and Han (2014) further suggested that in a variety of cultures, red is a sign of warning and caution as we see stop signs are colored red, and traffic lights indicating the color red means to stop. Red ink is often used to indicate errors in written work and overall red ink can be associated with negative behaviors (Zhang & Han, 2014). Past research on color showed that those wearing the color red in the Olympic games were more likely to win than their opponents wearing the color blue. It has been suggested that this is due to the fact that red is most often linked to characteristics like dominance and aggression (Hill & Barton, 2005). Meier, D’Agostino, Elliot, Maier, and Wilkowski (2012) indicated that the color red can be viewed as a threat or a sign of caution as well. They posited that red can also be viewed as a signal of romance and sex. Taylor, Schloss, Palmer, and Franklin (2013) found that most adults demonstrate a color preference and that blues are preferred most while yellows are least preferred.

There have been several theories related to what governs these preferences, including the ecological valence theory (EVT). According to Palmer and Schloss (2010), EVT proposes individual color preferences result from our own affective responses to color-associated objects. In other words, people tend to like or dislike colors to the same degree that they like or dislike the objects that are most commonly associated with that color. EVT further suggests that with some limitations, color preferences can be learned with experiences and interactions throughout one’s lifetime (Taylor, Schloss, Palmer, & Franklin, 2013).

The impact of color on decision making can also be applied in areas such as sales and marketing. The use of color has been established as a major contributor to consumer choices and perceptions (Gorn, Chattopadhyay, Yi, & Dahl, 1997). Abril, Olazabal, and Cava (2009) found that color carries an intrinsic meaning that contributes to the identity and recognition of a particular brand. Labrecque and Milne (2012) examined how color impacts consumer perceptions and found that various brands use color to form relationships, establish visual identity and even position themselves as lead competitors. Major corporations like Coca-Cola and Pepsi use color to distinguish themselves from their competitors; Coca-Cola using the color red and Pepsi embracing the color blue. The use of color can be also extended beyond the sale of products too as the Susan G. Komen Breast Cancer Foundation relies on the color pink to increase awareness. Clearly color psychology topics are important research areas.

Research has demonstrated that the color red can influence psychological functioning and this red effect can be observed in a variety of settings. The current literature also suggests that there is a link between color and perceived attractiveness of the subject to a viewer (Maier et al., 2013). The potential impact of the color red on attractiveness to other life factors has not been determined. There have been previous studies on attractiveness and the effect of color, yet none of those studies has applied the influence of the color red on attractiveness to other aspects of life, such as healthiness. By gaining a better understanding of the impact of color on individual perception, we may learn more about how color and healthiness are related to the assessment of attractiveness. The present research examined the effect the color red has on influencing perceived facial attractiveness to the perceived healthiness of females while they are described as eating healthy or unhealthy foods.

## 2. Method

An experimental design was developed to test the following research question: Does the color red impact the perception of facial attractiveness and perceived healthiness, regardless of what food a person is eating? It was hypothesized that a female wearing a red shirt will increase her perceived facial attractiveness and perceived healthiness, and that the food she is eating will moderate the effect.

### 2.1 Participants

All participants were adults who signed a written informed consent. The St. Bonaventure University Institutional Review Board (IRB) approved the protocol for this study. Participants were 122 undergraduate college students (54 males, 68 females) who were recruited using university classroom visits and sign-up sheets. Participant characteristics by gender are given in Table 1.

**Table 1:** Description of participant characteristics by gender. Age (years), weight (kg), height (cm), and BMI (kg/m^2^)

Variables	Age(SD)	Weight (SD)	Height(SD)	BMI(SD)
Male	19.85(1.85)	71(15.98)	170.56(13.00)	24.52 (4.42)
Female	19.88(1.44)	65(12.51)	164.46(12.57)	24.09 (3.70)
Totals	19.87(1.63)	67(14.44)	167.16(13.08)	24.28 (4.02)

### 2.2 Measures

**Figure 1:**
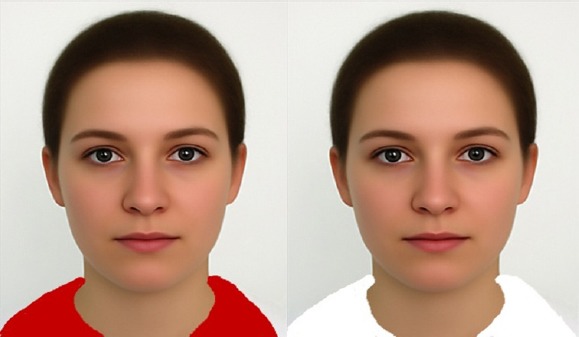
A sample image for each color manipulation. In each image, the female illustrated was the same; only the color of her shirt differed (red or white)

Participants completed a survey packet containing 4 images of the same female wearing either a red or a white shirt. The only difference in the images was the color of the shirt that the female was wearing (red or white). White in this case was used as the ‘neutral’ color. A sample of each image is given in Figure 1. Each image had an attached vignette in which the female was described as eating a healthy food or an unhealthy food. The healthy food vignette stated: Anna had an hour break for lunch. She decided to purchase a fresh salad from the local café. The unhealthy food vignette stated: Anna had an hour break for lunch. She decided to go to McDonald’s, where she purchased a double cheeseburger and French fries. Each vignette was included for the female wearing the red shirt (red-healthy; red-unhealthy), and for the female wearing the white shirt (white-healthy; white- unhealthy).

### 2.3 Procedure

The current study consisted of a 2 × 2 within-subjects factorial design. A within-subjects design was used to enhance the power of the study. The independent variables were the color of the shirt that the female image was wearing (red, white) and the type of food choice presented in the vignette (healthy vs. unhealthy). Ratings of perceived attractiveness and healthiness were recorded for each image on two 7-point rating scales. The attractiveness scale ranged from 1 (not attractive) to 7 (very attractive). The healthiness scale ranged from 1 (very unhealthy) to 7 (very healthy). A Latin Square was used to counterbalance the order that the images were presented to participants, so that the order was randomized across participants. After all of the images were viewed and rated on the accompanying rating scales, each participant was given a debriefing form and dismissed.

### 2.4 Statistical Analysis

Data were analyzed using a 2 × 2 within-subjects factorial analysis of variance (ANOVA). The within-subjects factors were shirt color (red, white), and the vignette (healthy, unhealthy). The dependent variables were attractiveness ratings, and healthiness ratings. Planned comparison related samples *t* tests were computed for significant interactions, and a Bonferroni correction procedure was used to control for experiment-wise alpha. Because each factor had only two levels, main effect tests were not needed. All statistical tests were computed at a.05 level of significance.

## 3. Results

**Figure 2:**
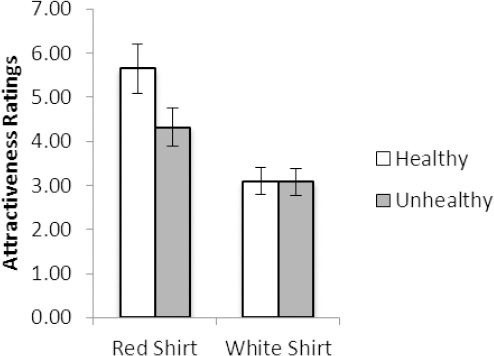
Attractiveness ratings show evidence of the “red effect” with high attractiveness ratings for the female that wore a red shirt. The “red effect” was evident when the female was described as eating a healthy food, but not when described as eating an unhealthy food

For attractiveness ratings, a significant main effect of shirt color was evident, F(1, 121) = 13.48, *p* <.001 (*R*^2^ =.10), with attractiveness ratings (*M*±SD) being significantly higher for the female wearing the red shirt (4.6±1.8) versus a white shirt (3.0±1.2). As shown in Figure 2, a significant shirt color × vignette interaction was also evident, F(1, 121) = 13.09, *p* <.001 (*R*^2^ =.10). Planned comparison related samples *t* tests showed a “red effect” for the female described as eating a healthy food, *t* (121) = 6.84, *p* <.001 (*d* = 0.62), but not for the female described as eating an unhealthy food, *p* =.80. Thus, ratings of attractiveness were significantly higher for the female described as eating a healthy food wearing a red (5.3±1.6) versus white (3.1±1.1) shirt. However, there was no significant difference in attractiveness ratings for the female described as eating an unhealthy food wearing a red (3.9±2.1) versus white (2.9±1.4) shirt.

**Figure 3:**
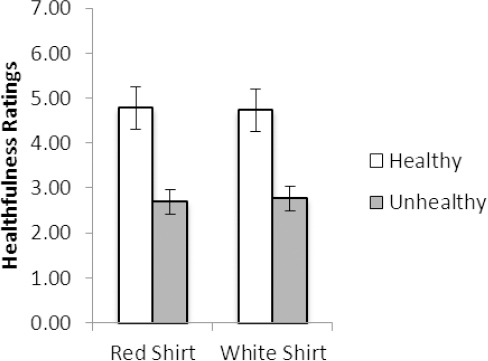
Healthiness ratings show that the female who was described as eating the healthy food was rated as healthier, regardless of the shirt color

For healthiness ratings, only a significant main effect of vignette was evident, F(1, 121) = 86.06, *p* <.001 (*R*^2^ =.42), with healthiness ratings (*M*±SD) being significantly higher for the female described as eating the healthy (4.5±1.4) versus unhealthy (2.9±1.4) food. Figure 3 displays the data for healthiness ratings by vignette type.

## 4. Discussion

The purpose of this investigation was to examine the effect the color red has on influencing perceived facial attractiveness to the perceived healthiness of females while they are described as eating healthy or unhealthy foods. This study posited that that a female wearing a red shirt will increase her perceived facial attractiveness and perceived healthiness, and that the food she is eating will moderate the effect. The findings show that the red shirt did increase the perceived attractiveness of a female, regardless of the food that she was eating. These data support the hypothesis tested and indicate that a red effect was found to be present as it relates to attractiveness. For healthiness, ratings show that the female who was described as eating the healthy food was rated as healthier, regardless of the shirt color, thus no red effect was identified in the present study related to healthiness.

The perception and association of a specific color as positive or negative is subjective, and yet research findings tend to support the idea that there is a red effect present when making evaluative decisions. By identifying a color that evokes more positive responses and feelings related to attractiveness, we can more accurately predict how the greater population will respond to color choices, and include the color red in areas where we want the perception of desirability. This red effect can be applied in all areas of our lives, and the color red can influence our individual decision making and responses to others.

There are however, several limitations that can be identified in this research. First, the research was conducted in a relatively small geographic area which may not represent the larger population. Second, the number of participants in the research was small and consisted of college students only who were all relatively close in age, thereby minimizing generalizability to larger and older populations. Third, the case vignettes were designed by the researcher for the purpose of this study alone, so the validity and reliability of the responses were not established beyond face validity. Finally, no demographic data were collected related to ethnicity and culture, so we cannot identify if there are any cultural implications or limitations in this study. In light of these limitations, for future research outreach to a larger number of ethnically and culturally diverse participants across a variety of ages within a wider geographic area would be warranted to obtain a larger and more representative sample.

## 5. Conclusion

Colors carry multiple meanings and the context may impact the associations that are impacted in memory. The central premise of this research was that females wearing a red shirt will increase her perceived facial attractiveness and perceived healthiness, and that the food she is eating will moderate the effect. For attractiveness ratings, a significant main effect of shirt color was evident. For healthiness ratings, only a significant main effect of vignette was evident. These findings contribute to the emerging literature on the impact of color on perceived attractiveness. In light of the findings of the present study, the red effect was found to be present and results indicated that color can have an effect on the identification of a female as attractive. Color can be used as a means of communication. The use of color can send a positive or negative message, and these findings contribute to the emerging literature on color psychology, as well as how the color red can influence our thoughts and opinions related to perceived attractiveness. Future studies on the impact of color on attractiveness and healthiness could provide additional information about this relationship.
